# Identification of *BRD4* as a Shared Diagnostic Biomarker Between Bipolar Disorder and Multiple Sclerosis Using Machine Learning

**DOI:** 10.3390/cimb48101077

**Published:** 2026-10-19

**Authors:** Xiaoyuan Zhou, Yixuan Zhang, Youzhi Xu, Chunyue Huo

**Affiliations:** College of Clinical Medicine, Capital Medical University Yanjing Medical College, Yanqin Building Room 207, Da dong Road 4, Shunyi District, Beijing 101300, China

**Keywords:** bipolar disorder, multiple sclerosis, *BRD4*, machine learning, biomarker, neuroinflammation

## Abstract

Bipolar disorder (BD) is closely associated with multiple sclerosis (MS), but their shared molecular mechanisms and specific diagnostic biomarkers remain unclear. This study integrated microarray expression profiling and machine learning to identify shared core genes and underlying mechanisms between BD and MS. Gene expression datasets and peripheral-blood transcriptome datasets were obtained from the GEO database. After per-cohort quantile normalization, shared genes were identified by differential expression analysis and weighted gene co-expression network analysis. A protein–protein interaction network was constructed, and key genes were screened using the MCODE plugin. Three machine learning algorithms were combined to identify the final core gene. A total of 229 shared genes were initially obtained and further filtered to 55 key genes. *BRD4* was identified as the final shared core gene. Discriminative performance (AUC, 95% CI) was: BD discovery GSE23848 (20 cases/15 controls) 0.713 (0.54–0.89); MS discovery GSE21942 (12/15) 0.998 (0.99–1.00); BD external GSE39653 (8/24) 0.775 (0.59–0.96); MS external GSE203241 (38/21) 0.828 (0.71–0.93). *BRD4* shows moderate-to-good discriminative performance as a non-invasive peripheral biomarker and may aid further investigation of shared BD–MS mechanisms.

## 1. Introduction

Bipolar disorder (BD) is a common severe mental illness characterized by alternating manic and depressive episodes [1], with a global lifetime prevalence of approximately 1–2% [2]. Twin and family studies have further estimated its heritability at around 80% [3], pointing to a substantial genetic contribution. In China, patients with BD bear a heavy disease burden [4]. Its pathogenesis is closely related to neurotransmitter imbalance, neuroinflammation, and abnormal epigenetic regulation, and it is often comorbid with autoimmune diseases [5,6]. In addition, patients with bipolar disorder show variable courses, which often lead to cognitive impairment and significantly reduced quality of life, and tend to get worse as the disease progresses [7]. Notably, BD has the highest suicide rate among all mental illnesses, with a suicide risk 20–30 times that of the general population [8]. However, the clinical diagnosis of BD faces significant challenges—due to symptom overlap with other mental disorders or autoimmune diseases, over half of clinicians may fail to make an accurate diagnosis, leading to undertreatment or inappropriate treatment that not only fails to alleviate symptoms but may also worsen the condition [9].

Multiple sclerosis (MS) is an autoimmune disease that causes inflammatory demyelinating lesions of white matter in the central nervous system [10], with a global prevalence of approximately 30 per 100,000, and its incidence in China is increasing annually [11]. Even though the cause of MS is unclear, current findings suggest that environmental and genetic variables contribute to the disease’s development [12]. Several environmental factors, such as infection, latitude, vitamin D deficiency, and smoking [13], contribute to the development of MS. Notably, MS patients often have comorbid mood disorders. Intriguingly, diffusion-tensor MRI studies have reported comparable white-matter microstructural alterations in BD, a feature proposed as an endophenotype that may link BD to the white-matter pathology of MS [14]. Epidemiological evidence indicates a significantly elevated risk of bipolar disorder (BD) in individuals with multiple sclerosis (MS). A systematic review reports a lifetime BD prevalence as high as 8.4% in MS patients [15], substantially exceeding the rate in the general population (<1%). This suggests potential shared underlying mechanisms between the two conditions. However, the molecular mechanisms and pathological interactions between the two remain unclear.

Recent research indicates that immune dysregulation and neuroinflammation are key links in the comorbidity of BD and MS: genome-wide association studies have also implicated the MHC region in both schizophrenia and BD, suggesting a shared immune-genetic basis with MS [16]. Levels of pro-inflammatory cytokines (e.g., IL-6, TNF-α) are elevated in the peripheral blood of BD patients, while the autoimmune process in MS also relies on immune attacks mediated by inflammatory factors [17,18,19]. Furthermore, both disorders exhibit abnormal oxidative stress and neural cell metabolic disturbances, suggesting potential overlap at the gene expression level [20,21]. However, the core genes driving the comorbidity of these two diseases and their specific regulatory mechanisms remain unclear, limiting the development of targeted diagnostic and therapeutic strategies.

As microarray and high-throughput sequencing technologies continue to advance quickly, bioinformatics techniques are frequently used to investigate the crosstalk between diseases in order to reveal the connections between the cellular and molecular mechanisms of diseases. Weighted Gene Co-expression Network Analysis (WGCNA) can identify key modules related to phenotypes through gene co-expression patterns, overcoming limitations of single DEG screening. Previous studies have mostly focused on the mechanisms of single diseases; reports systematically screening shared core genes between the two disorders using multi-omics and multi-algorithm integration strategies are scarce. In this study, we explored shared transcriptomic crosstalk genes between BD and MS using an integrated multi-algorithm bioinformatics framework, with the aim of identifying robust peripheral-blood biomarkers.

## 2. Materials and Methods

### 2.1. Data Collection and Preprocessing

Gene expression microarray datasets for BD and MS were downloaded from the GEO database (https://www.ncbi.nlm.nih.gov/geo/, accessed on 15 July 2025). Detailed dataset information is presented in Table 1. The following inclusion criteria were applied to ensure data reliability and representativeness: (1) datasets included both case and healthy control groups; (2) sample types were consistent with the research focus (peripheral blood or peripheral blood mononuclear cells [PBMCs]), facilitating potential clinical sample validation; (3) datasets provided complete gene expression matrices and sample annotation information; (4) dataset platforms were widely used with good data quality. Probe IDs were mapped to unique gene symbols via GPL annotation files. For each dataset, raw Affymetrix CEL files were background-corrected, quantile-normalized and log2-transformed using the standard limma pipeline; because each cohort was analyzed as an independent case–control contrast, no cross-dataset batch correction was required, and all downstream analyses were performed cohort by cohort without pooling data across platforms. All expression matrices were archived as CSV files for reproducibility.

### 2.2. Identification of Differentially Expressed Genes (DEGs)

We normalized raw expression matrices in R and identified DEGs of GSE23848 (BD) and GSE21942 (MS) via the R package “limma” with an empirical Bayes three-step pipeline. Because the two disorders differ in the magnitude of their peripheral-blood transcriptomic footprints, we applied disease-specific fold-change cutoffs while retaining a consistent nominal significance threshold of *p* < 0.05: |log_2_FC| > 0.1 for BD, in which peripheral immune changes are relatively subtle and we wished to retain weakly expressed but biologically relevant immune transcripts; and |log_2_FC| > 0.5 for MS, in which pronounced autoimmune activation produces large expression shifts, so a higher cutoff was used to filter trivial noise. The final candidate *BRD4* itself was highly robust, surviving Benjamini–Hochberg multiple-testing correction in the MS discovery cohort. As *BRD4* itself survived Benjamini–Hochberg correction in the MS discovery cohort, the identification of the final biomarker is not sensitive to the choice of nominal versus adjusted *p*-value. Variable-gene heatmaps and global DE patterns were visualized using the pheatmap and ggplot2 packages.

### 2.3. Weighted Gene Co-Expression Network Analysis (WGCNA)

The co-expression networks for BD (GSE23848) and MS (GSE21942) were constructed using the R package “WGCNA” in R. The top 25% of genes with the highest variance were selected to construct a scale-free co-expression network. Outlying samples were first identified by average-linkage hierarchical clustering; one outlier was removed from the BD network, and no outlier was detected in the MS network. A soft-threshold power β = 8 was adopted to construct unsigned scale-free networks (BD: scale-free R^2^ = 0.943; MS: R^2^ = 0.852), with minModuleSize = 100 and modules merged at a cut height of 0.25. Module eigengenes were correlated with phenotypic traits using Pearson’s correlation, and a heatmap was generated to visualize the module–trait relationships. Disease-associated modules were defined as those with |module–trait r| ≥ 0.5 and *p* < 0.01. Gene lists of all significant modules were exported for cross-disease intersection analysis.

### 2.4. Identification of Shared Genes and Pathway Enrichment

By drawing Venn diagrams, the shared genes identified by WGCNA and DEG were obtained. Genes overlapping BD DEGs, MS DEGs and their respective disease-related modules were extracted using nested intersect functions. Venn plots were generated via the ggvenn R package. Then, we explored functions and pathways associated with these genes through Gene Ontology (GO) and Kyoto Encyclopedia of Genes and Genomes (KEGG) using “clusterProfiler” and “org.Hs.eg.db” packages. Gene symbols were converted to Entrez IDs prior to enrichment analysis, with Benjamini–Hochberg (BH) correction adopted for multiple testing. Top 10 enriched terms of each GO category and top 25 KEGG pathways were visualized via bar and dot plots.

### 2.5. Construction of Protein–Protein Interaction (PPI) Network and Screening of Key Genes

The initially screened core genes were uploaded to the STRING database (https://string-db.org/, accessed on 28 July 2025), with the species set to “Homo sapiens” and the confidence threshold set to 0.4. The MCODE plugin in Cytoscape (version 3.8.2) was used to screen key gene modules within the PPI network. Genes within the key modules were defined as key genes, and the node degree for each key gene was calculated, visually reflecting gene importance.

### 2.6. Machine Learning Screening for Core Genes

To further screen genes with high diagnostic value from the key genes, three machine learning algorithms were used for cross-validation and screening, and the intersection of the results was taken to determine the final core genes. Least absolute shrinkage and selection operator (LASSO) yields a parsimonious and interpretable sparse set, Random Forest captures non-linear and interaction effects via Gini importance, and support-vector-machine recursive feature elimination (SVM-RFE) wraps a maximum-margin classifier around recursive feature elimination. Requiring genes to be selected by all three methods reduces dependence on any single algorithm’s bias and improves robustness. All feature selection and hyperparameter tuning were performed entirely within the cross-validation folds on the discovery cohort, and the external validation cohorts were not used at any stage of feature selection or model training. These three packages are among the most widely used feature-selection tools in transcriptomics; requiring their intersection is a standard cross-algorithm consensus filter that reduces reliance on any single method’s bias.

The R package “glmnet” was used for LASSO regression analysis on the key genes. The optimal λ value was determined via 5-fold cross-validation, using mean squared error (MSE) as the evaluation criterion (the default Gaussian loss in glmnet for continuous outcomes), and both minimum lambda (λ.min) and one standard error lambda (λ.1se) were selected as the optimal cutoffs, and genes with non-zero regression coefficients were selected as candidate genes.

The R package “randomForest” was used to build a Random Forest model, with the number of decision trees set to 500. Gene importance was calculated based on the Gini index, and genes with a Gini importance value greater than 0.5 were selected as candidate genes. This threshold was chosen to prioritize genes with a non-negligible contribution to classification while retaining a manageable candidate set.

The R package “e1071” was used for SVM-RFE analysis. Model classification accuracy after removing each feature was evaluated via 5-fold cross-validation, and the optimal gene subset was determined based on the minimum cross-validation error rate, and genes significantly contributing to the classification boundary were selected as candidate genes.

Venn diagrams were drawn to show the intersection of candidate genes screened by the three algorithms, and the intersecting genes were defined as the final core genes for BD and MS. *BRD4* was the sole gene identified by all three algorithms in both BD and MS cohorts, and was therefore selected as the shared candidate for subsequent correlation, diagnostic and nomogram analyses.

### 2.7. Validation of Core Gene Expression and Analysis of Diagnostic Efficacy

Boxplots were drawn using the “ggplot2” package to compare the expression differences in the core gene between case and control groups. Independent samples *t*-test was used to test the statistical significance of expression differences.

The R package “pROC” was used to generate ROC curves, with *BRD4* expression level as the sole predictor, for the core gene in the training sets (GSE23848, GSE21942) and external validation sets (GSE39653, GSE203241), and the Area Under the Curve (AUC) was calculated. The Bootstrap method (1000 resamples) was used to calculate the 95% confidence interval (CI) for the AUC, and the optimal diagnostic threshold was determined by the Youden index. The discriminative performance of *BRD4* across the four cohorts is summarized in Table S2.

### 2.8. Immune Cell Infiltration and BRD4 Correlation Analysis

The CIBERSORT algorithm was applied to quantify the proportions of immune cell types using the LM22 signature matrix, with 1000 permutations and quantile normalization enabled. Samples with CIBERSORT *p* < 0.05 were retained for downstream analysis. Wilcoxon rank-sum tests were used to compare immune cell fractions between patients and healthy controls, and Pearson correlation analysis was performed to evaluate associations between *BRD4* expression and individual immune cell subsets. Nominal *p* < 0.05 was used as the significance threshold, and the strongest associations (e.g., naive B cells in MS, *p* = 1.9 × 10^−5^; *BRD4*–naive B cells, *p* = 9.0 × 10^−9^) would survive Benjamini–Hochberg correction across the 22 cell types. All visual outputs, including immune infiltration histograms and correlation bar plots, were generated using R packages.

### 2.9. Construction and Validation of the BRD4-Based Diagnostic Nomogram

Univariate diagnostic nomograms incorporating *BRD4* as the only predictor were constructed for each dataset using the “rms” R package. Calibration curves and decision curve analysis (DCA) were subsequently adopted to systematically assess the predictive consistency and net clinical benefit of the risk model.

### 2.10. Statistical Analysis

All statistical analyses were performed using R 4.5.1 (R Foundation for Statistical Computing, Vienna, Austria; packages: limma, WGCNA, glmnet, pROC, rms). The significance level was set to *p* < 0.05 for all tests.

## 3. Results

### 3.1. Identification of Differentially Expressed Genes (DEGs)

**Figure 1:**
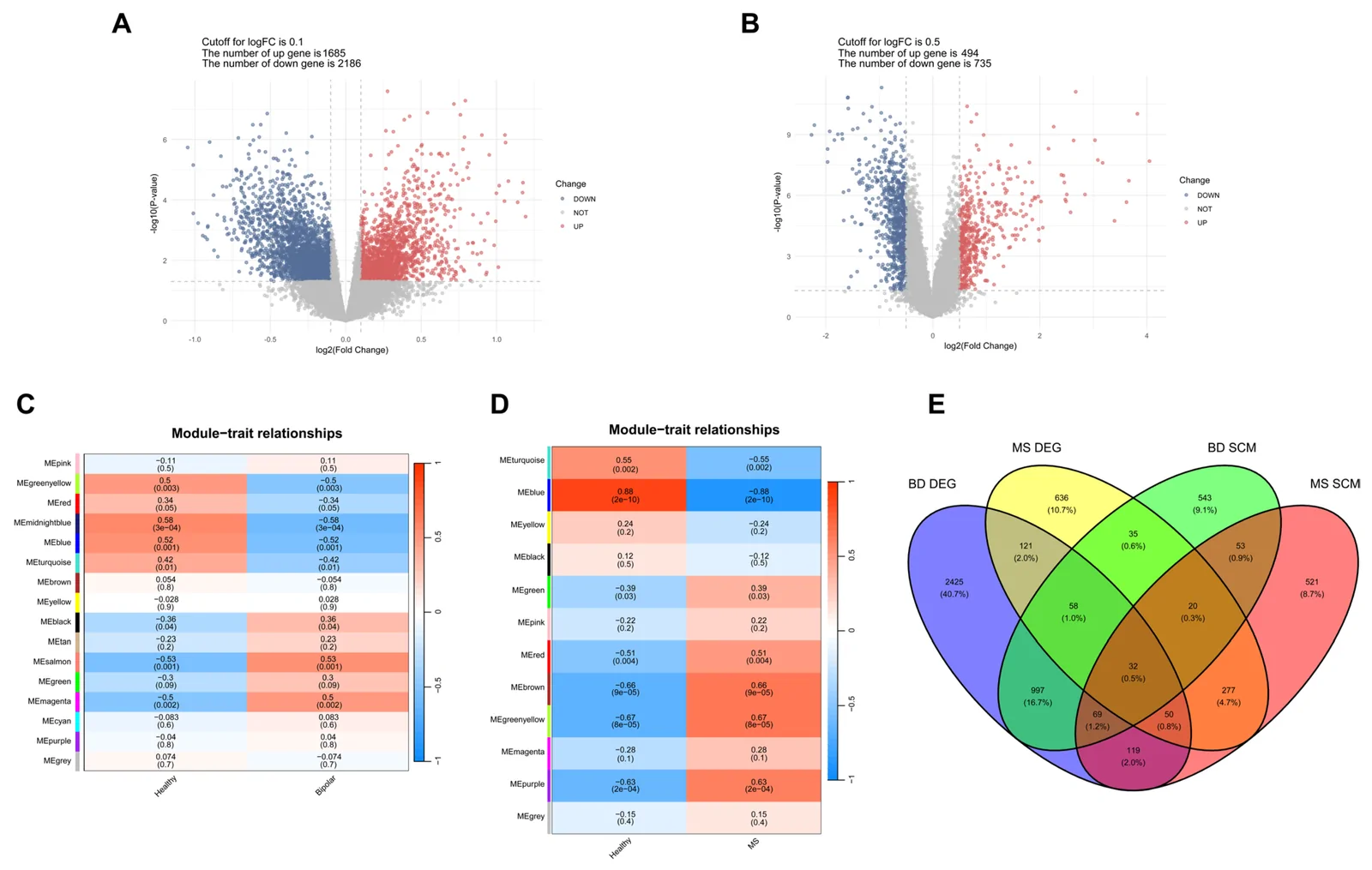
Volcano plots, WGCNA module-trait heatmaps and Venn diagram for BD and MS. (**A**) BD DEG volcano (GSE23848, |log_2_FC| > 0.1, *p* < 0.05); (**B**) MS DEG volcano (GSE21942, |log_2_FC| > 0.5, *p* < 0.05). Red = upregulated, blue = downregulated, gray = non-significant genes. X: log_2_FC, Y: -log_10_(adjusted *p*). (**C**,**D**) Module-trait heatmaps for BD and MS; cell values show correlation coefficients with *p*-values, darker colors indicate stronger correlation. (**E**) Four-set Venn diagram identifying 229 shared core genes at the intersection of BD DEGs, BD modules, MS DEGs and MS modules. Numbers represent gene counts, with percentages in parentheses.

After data standardization and LIMMA analysis, DEGs for BD and MS were obtained. The BD dataset (GSE23848) identified 3871 DEGs, including 1685 upregulated and 2186 downregulated genes, out of 24,999 tested genes (15.5%). The MS dataset (GSE21942) identified 1229 DEGs, including 494 upregulated and 735 downregulated genes, out of 22,881 tested genes (5.4%). The higher number of DEGs in BD reflects the more permissive |log_2_FC| > 0.1 threshold used to retain subtle peripheral-blood transcripts. Volcano plots showed distinct distribution of DEGs in both diseases, with upregulated DEGs clustered in the positive Log_2_FC region and downregulated DEGs clustered in the negative Log_2_FC region, indicating significant expression changes between case and control groups (Figure 1).

### 3.2. Screening of WGCNA Significant Modules

After constructing weighted gene co-expression networks, significant modules were identified through module-trait correlation analysis. The BD dataset (GSE23848) identified 16 co-expression modules (marked by different colors). The module-trait correlation heatmap showed that five modules were significantly correlated with the BD phenotype (|r| ≥ 0.5, *p* < 0.01): MEmidnightblue (r = 0.58, *p* = 3 × 10^−4^), MEsalmon (r = 0.53, *p* = 0.001), MEblue (r = −0.52, *p* = 0.001), MEgreenyellow (r = −0.50, *p* = 0.003), and MEmagenta (r = 0.50, *p* = 0.002). These five modules contained 1807 genes. The MS dataset (GSE21942) identified 12 co-expression modules. The MEblue module was selected as significantly correlated with the MS phenotype (r = 0.88, *p* < 2 × 10^−10^), indicating a close association with MS development; this module contained 1141 genes (Figure 1).

### 3.3. Acquisition of Initially Screened Core Genes

SCM genes represent the module genes obtained by weighted gene co-expression network analysis (WGCNA). Venn diagram analysis showed that the intersection of BD DEGs, BD SCM genes, MS DEGs, and MS SCM genes consisted of 229 genes, which were defined as the initially screened core genes for BD and MS. These genes are not only differentially expressed between case and control groups but also closely related to disease phenotypes, suggesting they may play important roles in the comorbidity of the two disorders (Figure 1 and Figure S1).

### 3.4. GO and KEGG Enrichment Analysis Results

**Figure 2:**
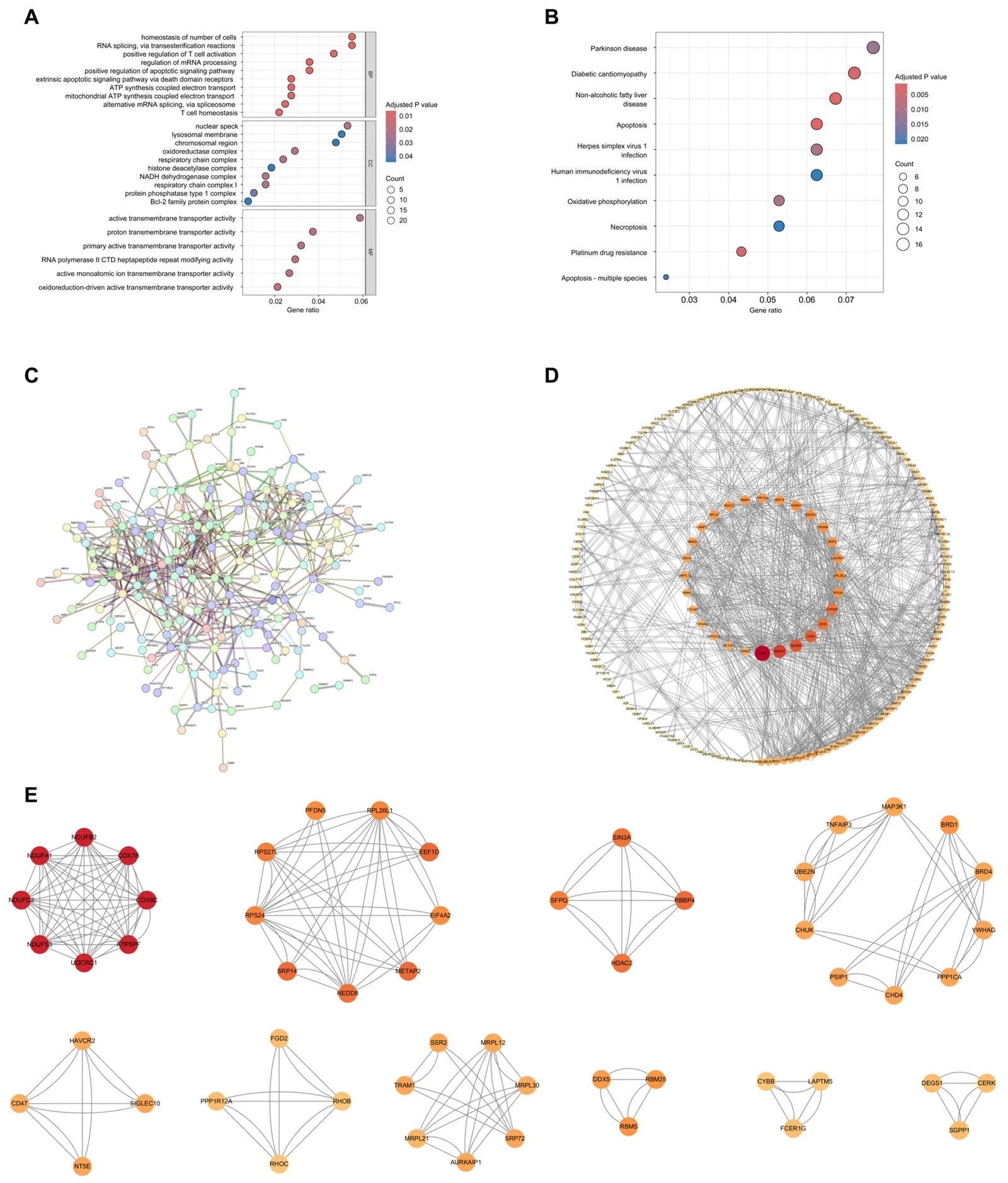
GO/KEGG enrichment and PPI network of the 229 shared genes. (**A**) GO bubble plot: bubble size = enriched gene number, color = adjusted *p*. (**B**) KEGG dot plot with identical color/size rules. (**C**,**D**) Full STRING PPI network of all shared genes. (**E**) MCODE-filtered core subnetwork containing 55 hub genes; larger nodes mean higher connectivity.

In the GO enrichment analysis, the top-ranked significantly enriched terms across categories were selected. Biological Processes (BP) were mainly enriched in “regulation of neurotransmitter metabolism”, “immune cell activation”, “response to oxidative stress”, and “regulation of apoptosis”; Cellular Components (CC) were mainly enriched in “postsynaptic membrane”, “immune synapse”, and “mitochondrial matrix”; Molecular Functions (MF) were mainly enriched in “cytokine binding”, “neurotransmitter receptor activity”, and “oxidoreductase activity” (Figure 2).

In the KEGG enrichment analysis, the 229 key genes were significantly enriched in 12 KEGG pathways. The top five pathways were “Oxidative phosphorylation”, “Huntington disease”, “Parkinson disease”, “Diabetic cardiomyopathy”, and “Human T-cell leukemia virus 1 infection” (Figure 2).

### 3.5. Results of PPI Key Genes

The 229 initially screened core genes were uploaded to the STRING database to construct a PPI network. After screening with the MCODE plugin, a key module containing 55 genes was obtained (Figure 2).

### 3.6. Results of Machine Learning-Based Core Gene Screening

**Figure 3:**
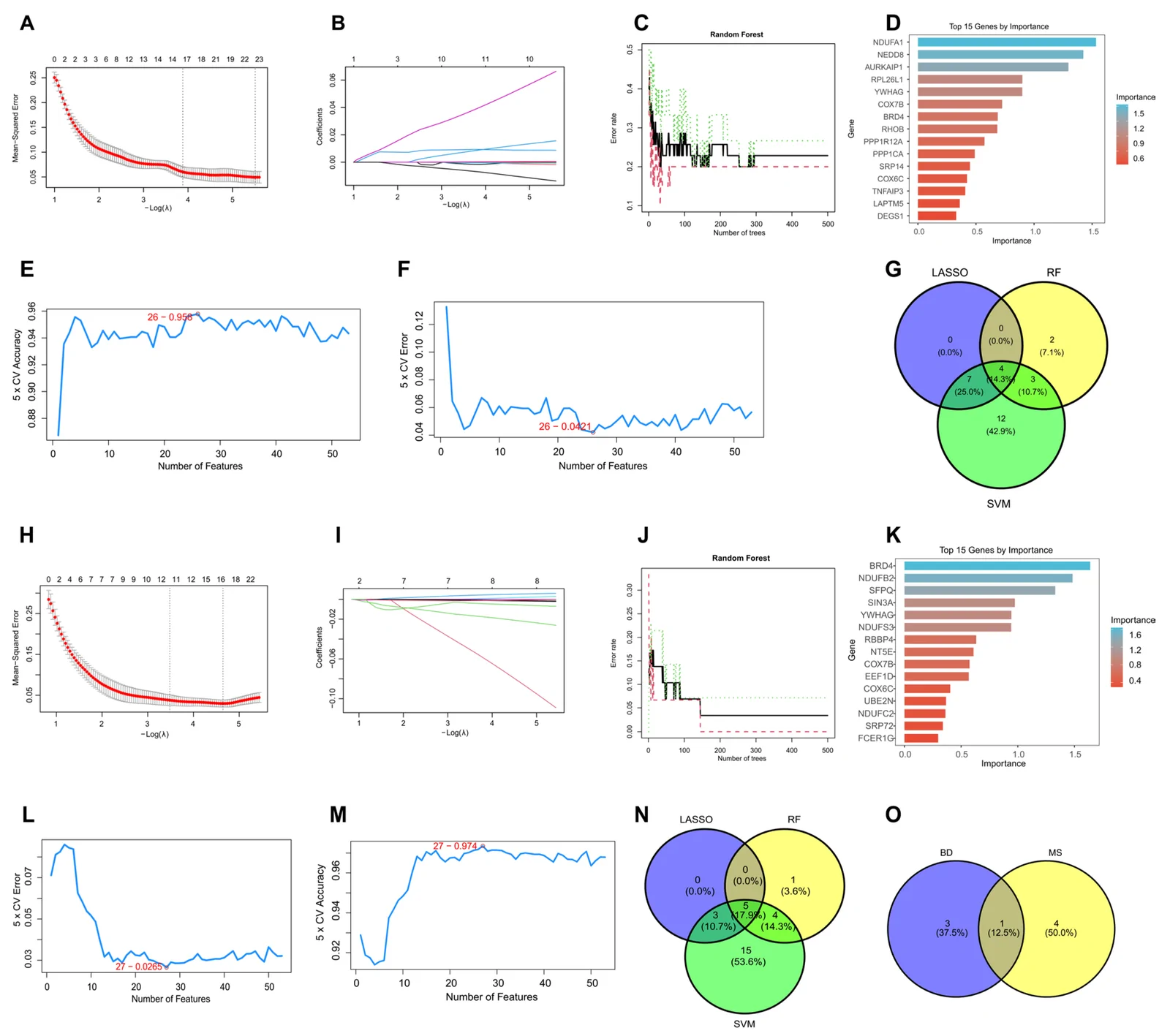
Machine learning screening of shared hub genes. Panels (**A**–**G**) display results from BD dataset GSE23848, including LASSO cross-validation and coefficient curves, Random Forest error and importance ranking, SVM-RFE accuracy/error curves, and three-algorithm Venn plot. Panels (**H**–**N**) show matching analyses for MS dataset GSE21942. (**O**) Cross-disease Venn plot confirms *BRD4* as the only overlapping gene across all algorithms in both cohorts.

Three reproducible machine learning pipelines with fixed random seed 12 and three repeated training rounds were applied to screen candidate genes from the 55 core genes. Stratified 5-fold cross-validation was uniformly adopted to balance case–control proportions in all training and testing subsets. Using glmnet (v4.1-8), LASSO regression yielded 11 candidate genes for GSE23848 (BD) and 8 for GSE21942 (MS). Random Forest (RF) built with 500 decision trees via randomForest (v4.7-1.1) identified 9 and 10 candidate genes from the two datasets, respectively. SVM-RFE implemented using the e1071 (v1.7-13) package extracted 26 candidates for GSE23848 and 27 for GSE21942 (Figure 3, Table S1).

Separate Venn diagrams were plotted to extract overlapping gene candidates from the three algorithms for BD and MS cohorts individually. Four intersecting genes were obtained from the BD dataset, and five from the MS dataset. A cross-disease Venn plot further integrated these two gene pools. Ultimately, *BRD4* (Bromodomain Containing 4) was the sole gene shared across all three algorithms and both disease datasets, which was designated as the definitive shared core gene of BD and MS (Figure 3).

### 3.7. Validation of Core Gene BRD4 Expression and Diagnostic Efficacy

**Figure 4:**
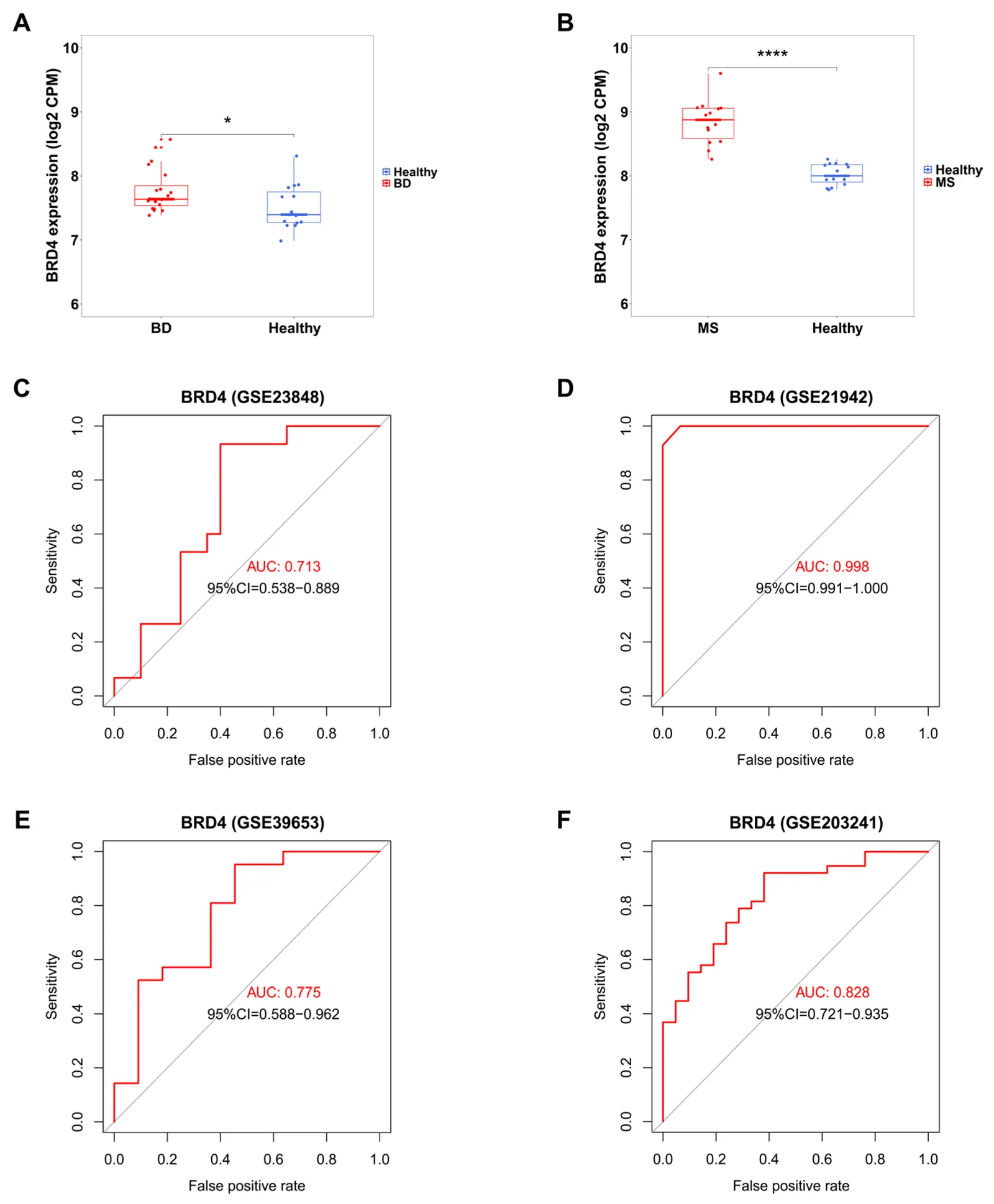
*BRD4* expression and ROC validation. (**A**) *BRD4* expression comparison between BD patients and controls (* *p* < 0.05). (**B**) *BRD4* expression comparison between MS patients and controls (**** *p* < 0.05). (**C**–**F**) ROC curves of *BRD4* in BD training, MS training, BD validation and MS validation cohorts, with AUC and 95% CI annotated on each plot. X: False positive rate, Y: Sensitivity.

Expression difference validation using boxplots showed that *BRD4* expression was significantly higher in BD patients (GSE23848) compared to healthy controls (*p* < 0.05); in MS patients (GSE21942), *BRD4* expression was extremely significantly higher than in healthy controls (*p* < 0.001). This indicates that *BRD4* is highly expressed in both BD and MS patients, with statistically significant expression differences (Figure 4).

Diagnostic efficacy analysis using ROC curves showed that *BRD4* achieved an AUC of 0.713 (95%CI: 0.538–0.889) in the BD training set (GSE23848) and an AUC of 0.998 (95%CI: 0.991–1.000) in the MS training set (GSE21942). *BRD4* achieved an AUC of 0.775 (95%CI: 0.588–0.962) in the BD external validation set (GSE39653) and an AUC of 0.828 (95%CI: 0.721–0.935) in the MS external validation set (GSE203241). As summarized in Table S2, the corresponding sensitivity and specificity at the optimal Youden threshold were 0.95/0.60, 1.00/0.93, 0.50/0.92 and 0.92/0.62, respectively (Table S2). Although the BD cohorts showed relatively wide 95% CIs (reflecting the smaller sample sizes), *BRD4* discriminated cases from controls in both discovery and independent validation cohorts, supporting its potential value as an auxiliary screening biomarker that warrants further validation in larger prospective cohorts (Figure 4).

### 3.8. Immune Infiltration Characteristics and BRD4 Immune Correlations

**Figure 5:**
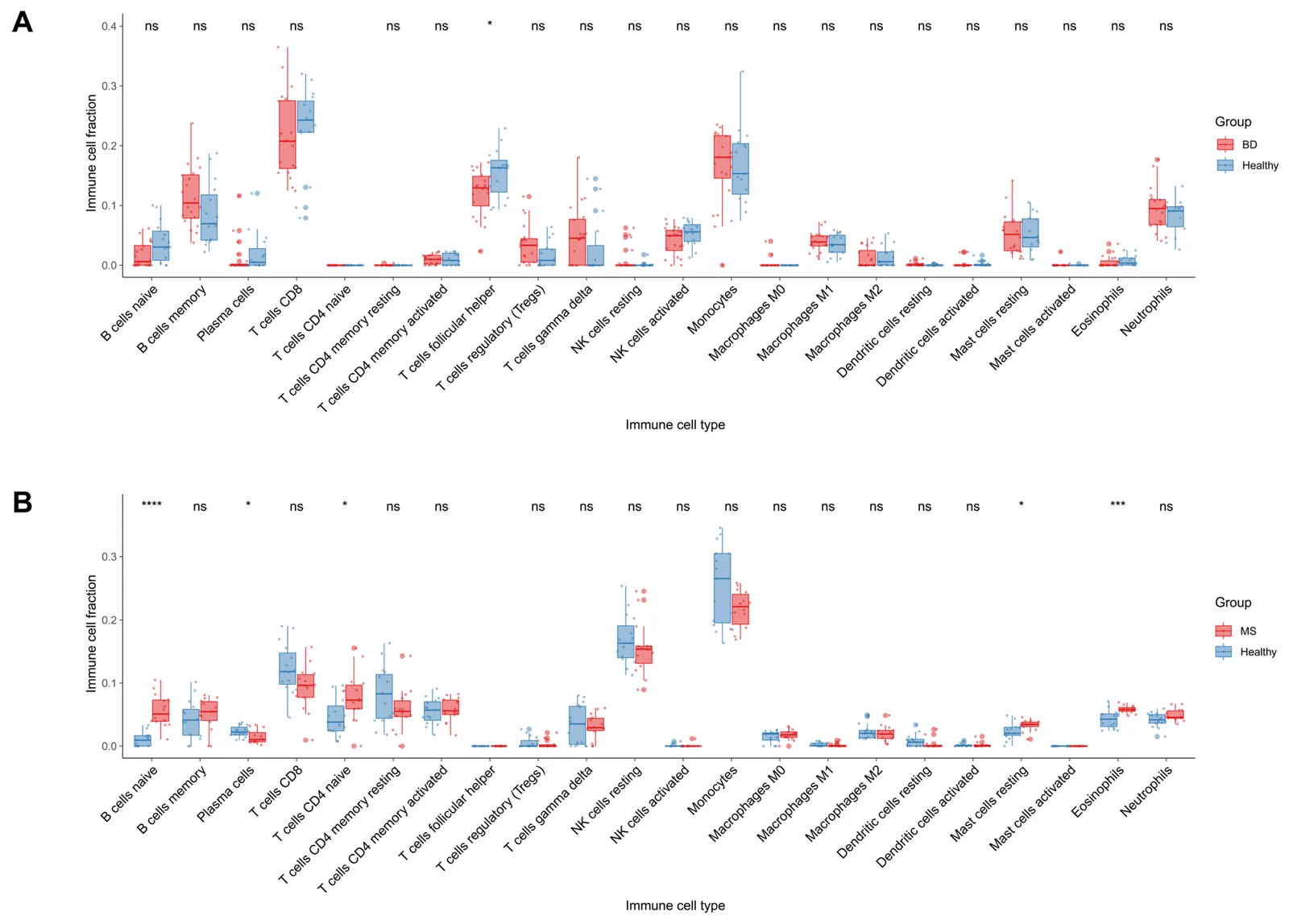
Immune infiltration. (**A**,**B**) CIBERSORT-derived immune cell proportions in BD and MS patients vs. controls. Blue = control, red = patient groups (* *p* < 0.05, *** *p* < 0.05, **** *p* < 0.05).

**Figure 6:**
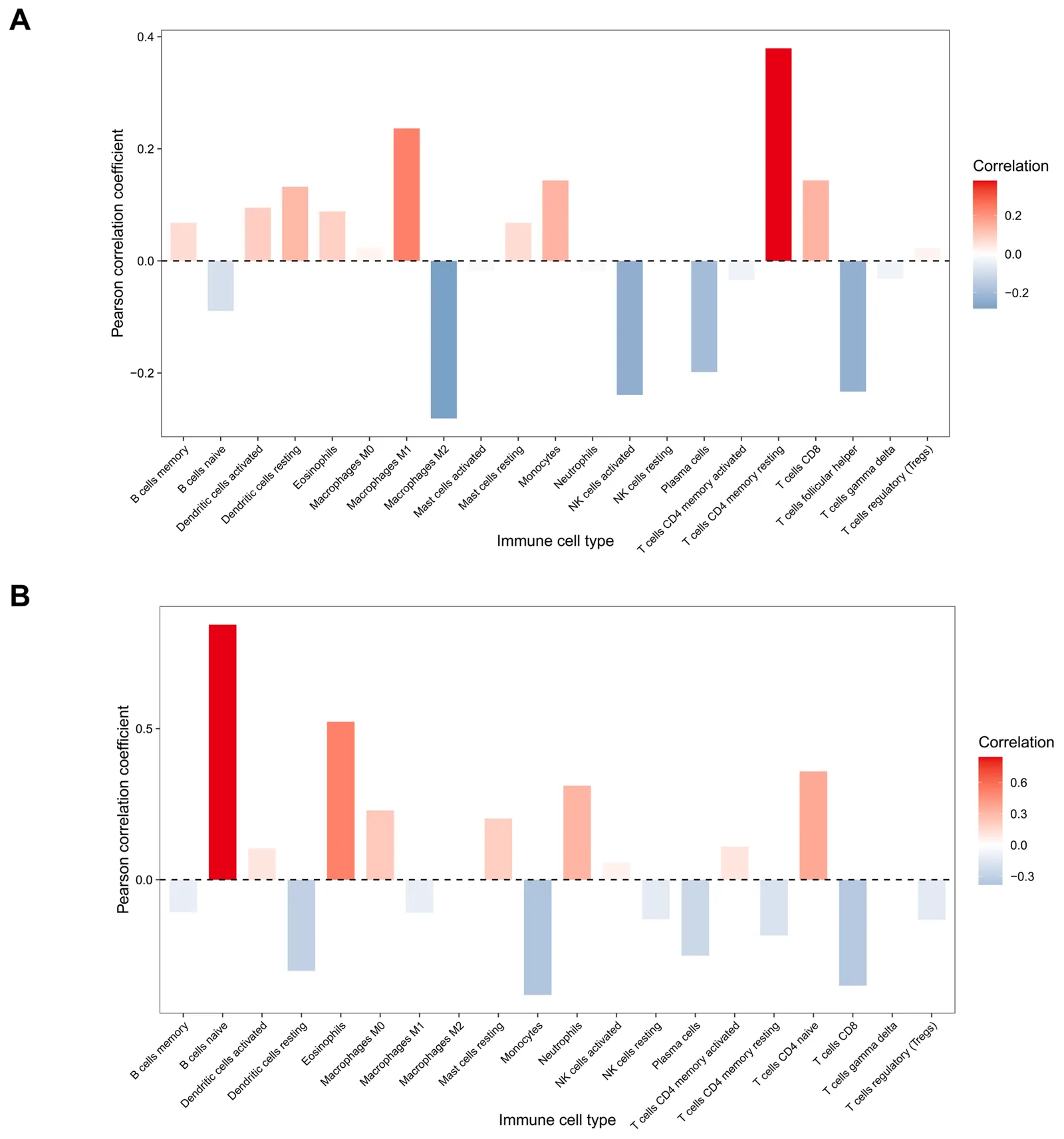
*BRD4*-immune correlations. (**A**,**B**) Pearson correlation bar charts between *BRD4* and each immune subtype; red bars = positive correlation, blue bars = negative correlation.

Differential patterns of immune disturbance were detected in BD and MS, with no consistently altered overlapping immune cell populations observed between the two disorders. In the BD cohort, T follicular helper cells were significantly reduced in patients (median 0.129 vs. 0.163, Wilcoxon *p* = 0.028), and *BRD4* expression was positively correlated with resting CD4 memory T cells (Pearson r = 0.38, *p* = 0.025). In the MS cohort, naive B cells (0.051 vs. 0.009, *p* = 1.9 × 10^−5^), eosinophils (0.058 vs. 0.043, *p* = 7.7 × 10^−4^), resting mast cells (0.035 vs. 0.020, *p* = 0.016) and naive CD4 T cells (0.073 vs. 0.038, *p* = 0.020) were elevated in patients, whereas plasma cells were reduced (0.010 vs. 0.022, *p* = 0.016). *BRD4* expression showed the strongest positive correlation with naive B cells (r = 0.84, *p* = 9.0 × 10^−9^), a moderate positive correlation with eosinophils (r = 0.52, *p* = 0.004), and an inverse correlation with monocytes (r = −0.38, *p* = 0.042) (Figure 5 and Figure 6, Table S3).

### 3.9. Performance Evaluation of the BRD4 Predictive Nomogram

**Figure 7:**
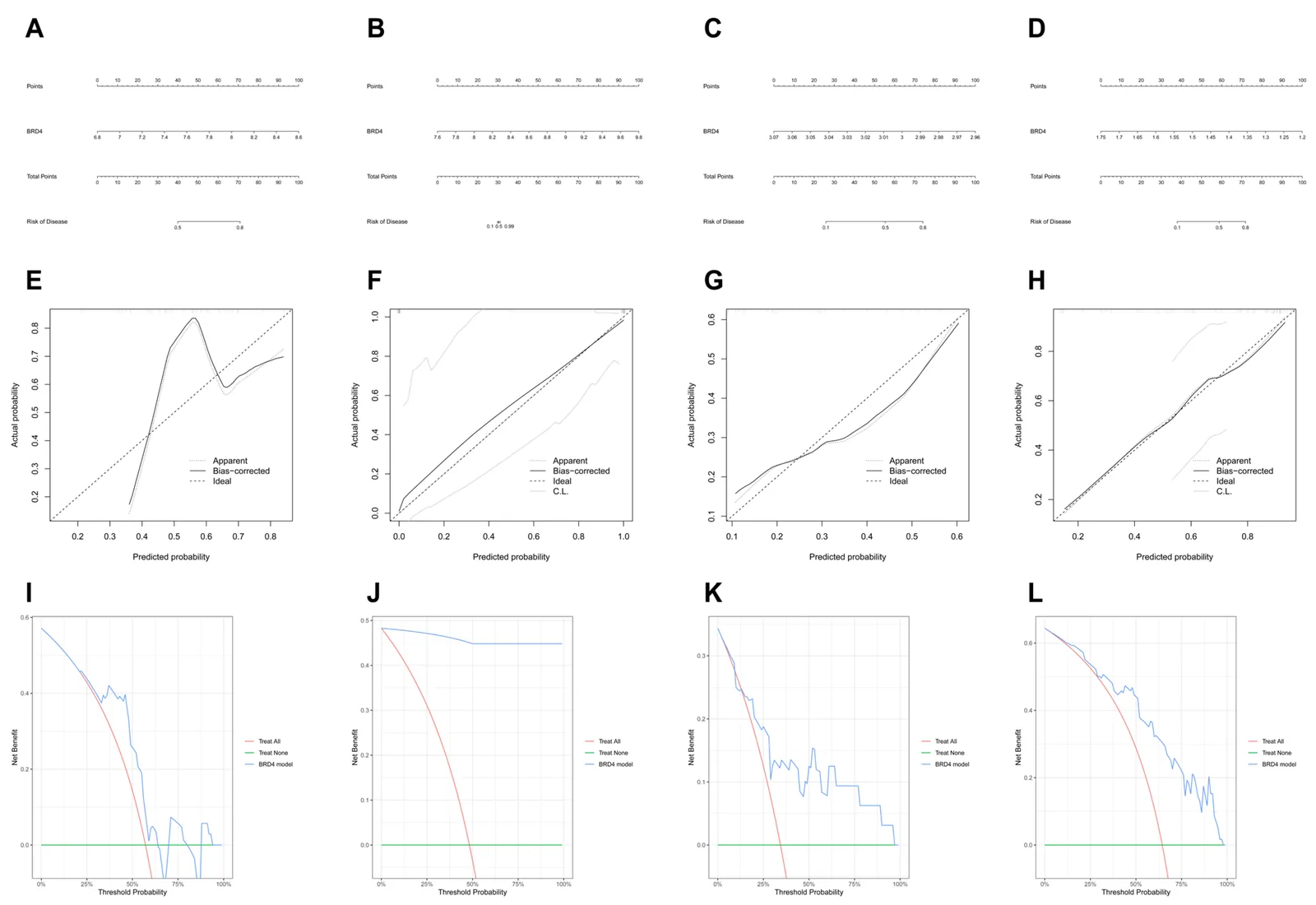
*BRD4*-based nomogram, calibration curves and DCA across four cohorts. (**A**–**D**) Univariate nomograms using *BRD4* to calculate disease risk scores. (**E**–**H**) Calibration curves; dashed diagonal line stands for ideal prediction. (**I**–**L**) DCA curves: blue = *BRD4* model, red = treat-all, green = treat-none. X: threshold probability, Y: net clinical benefit.

This single-gene nomogram allows intuitive individualized risk scoring for disease susceptibility. Calibration curves indicated satisfactory consistency between predicted and actual risks within MS cohorts, while slight predictive bias was detected in the BD training dataset. DCA revealed a broader range of net clinical benefit when utilizing *BRD4* for MS screening. By contrast, meaningful clinical utility was only observed in low-risk subgroups of patients with BD (Figure 7).

## 4. Discussion

Bipolar disorder (BD) and multiple sclerosis (MS) exhibit prominent clinical comorbidity [22]. Meta-analytic data reveal that MS patients carry an 8.4% lifetime risk of BD, far exceeding the prevalence in the general population, which strongly indicates overlapping molecular pathological cascades underlying the two disorders, and transcriptomic evidence has further confirmed convergent alterations in mitochondrial metabolism, oligodendrocyte homeostasis and systemic inflammatory signaling between BD and MS, yet systematic mining of shared diagnostic epigenetic biomarkers across both diseases remains insufficient [23]. Most existing transcriptomic studies separately investigate the independent pathogenesis of BD or MS, while systematic multi-algorithm screening for shared cross-disease diagnostic biomarkers remains rarely reported. This gap hinders accurate differential diagnosis and targeted intervention for patients with overlapping clinical manifestations. In the present study, we integrated peripheral blood transcriptomic profiles from the GEO database and constructed a multi-layer bioinformatic pipeline combining differential expression analysis, weighted gene co-expression network analysis (WGCNA), protein–protein interaction (PPI) clustering, and three complementary machine learning algorithms. After external cohort validation, we identified *BRD4* as the unique shared hub gene linking BD and MS, validated its stable cross-disease diagnostic performance, and proposed a *BRD4*-centered immune-neural crosstalk axis to interpret the molecular basis of their comorbidity, establishing a novel epigenetic framework connecting severe psychiatric illness and central nervous system autoimmunity [24]. *BRD4* (bromodomain-containing 4) is an epigenetic reader that recognizes acetylated histone tails via its tandem bromodomains and recruits the transcriptional elongation complex to target gene promoters, thereby coupling histone acetylation to gene transcription [25]. Because *BRD4* integrates signals from NF-κB, cAMP and other stress-responsive pathways, its expression can report on convergent inflammatory and neurochemical perturbations that are difficult to capture with a single cytokine marker. Its role as a convergent molecular node in both neuropsychiatric and autoimmune settings makes it a plausible candidate for further investigation.

Single-gene differential expression screening typically yields high false-positive rates [26], so we adopted a nested multi-step filtering strategy to improve screening reliability. First, we intersected disease-related differentially expressed genes (DEGs) and phenotype-correlated WGCNA modules from BD and MS datasets to obtain 229 shared genes, which simultaneously reflect transcriptional perturbation and coordinated co-expression patterns in both disorders. The WGCNA approach is particularly valuable here because genes that are not individually strong DEGs may still belong to a co-expression module tightly correlated with disease state; intersecting DEGs and module genes therefore captures both quantitative and network-level signals. The MCODE algorithm further clustered these genes from the PPI network and narrowed the candidate pool down to 55 functionally interactive core genes. To reduce bias from a single machine learning model, we integrated LASSO regression, Random Forest, and SVM-RFE for cross-screening [27]: LASSO removes redundant transcriptional noise, Random Forest quantifies gene discriminatory capacity via the Gini index, and SVM-RFE optimizes feature subsets through repeated 5-fold cross-validation. Extracting overlapping candidate genes across three algorithms effectively offsets model-specific deviation, which serves as a key methodological improvement compared with conventional single-algorithm workflows. The broader utility of machine-learning approaches in MS research is also supported by recent work identifying multidimensional predictors of quality of life in MS patients [28], underscoring the value of data-driven feature selection across MS outcomes. Prior research mostly excavated hub genes within BD or MS alone, with few studies focusing on shared epigenetic regulators for comorbidity [23]. Studies combining WGCNA and triple machine learning to mine cross-disease diagnostic markers are scarce, as single machine learning algorithms often generate biased feature selection results and fail to eliminate transcriptional noise from public microarray datasets [29]. Previous inflammation-focused work rarely identified *BRD4*—a BET family epigenetic transcription factor—as the central convergent mediator of neuropsychiatric and autoimmune injury [24]. Accordingly, our analysis fills a clear research gap regarding shared molecular markers underlying BD-MS comorbidity.

The GO and KEGG enrichment analyses converged on immune activation, oxidative stress and synaptic signaling as the shared axes of BD and MS, and *BRD4* occupied the most central position in the PPI network. Mechanistically, *BRD4* is not merely a passenger marker: as a bromodomain reader, it recognizes acetylated histones at active enhancers and recruits P-TEFb to release paused RNA polymerase II, thereby converting transient inflammatory signals into sustained transcriptional programs [25]. In the MS context, this program drives naive B-cell differentiation and germinal-center formation [30], which matches our observation that *BRD4* expression correlated most strongly with naive B cells (r = 0.84) and that naive B cells were the most significantly expanded population in MS patients. The same *BRD4*-dependent transcriptional machinery also operates in CD4+ T cells and microglia, where it amplifies NF-κB signaling and promotes CNS infiltration and demyelination in EAE models [24]. The BD side of the axis is less pronounced but directionally consistent. In BD peripheral blood, *BRD4* correlated with resting CD4 memory T cells rather than activated effector subsets, and only T follicular helper cells showed a modest reduction—consistent with the low-grade, chronic inflammatory profile long associated with mood disorders rather than the focal autoimmune attack seen in MS. At the neural level, *BRD4* in microglia and neurons regulates synaptic gene transcription and dendritic spine density [31]; sustained peripheral inflammation that elevates *BRD4* may therefore bias synaptic pruning and neurotransmitter homeostasis, providing a molecular route by which systemic immune activation could contribute to mood dysregulation. This dual peripheral–central engagement is the mechanistic link that makes *BRD4* a plausible shared node: in the periphery it reports on autoimmune B-cell activity in MS and low-grade T-cell shifts in BD, while centrally it may couple those inflammatory signals to the synaptic and neurochemical alterations common to both disorders.

ROC curve and single-gene nomogram analyses support the potential of *BRD4* as a minimally invasive auxiliary screening biomarker rather than a definitive diagnostic gold standard for BD and MS. In training datasets, *BRD4* achieved an AUC of 0.713 for BD and 0.998 for MS; external validation cohorts retained AUC values above 0.70 and 0.82, respectively, meeting the threshold for clinically meaningful predictive efficacy (AUC > 0.6). The superior predictive performance of *BRD4* in MS may be attributed to robust *BRD4*-dependent humoral autoimmune signaling, whereas mild and restricted T-cell inflammation in BD leads to narrower transcriptional variation and relatively weaker diagnostic power. Calibration curves demonstrated strong consistency between predicted and actual disease risk in MS cohorts, while mild predictive bias was observed in BD samples. This discrepancy may stem from limited sample sizes of public microarray datasets, unadjusted clinical confounders (including medication history and disease duration), and the inherent limitation that peripheral blood transcriptomics cannot fully recapitulate transcriptional alterations in central nervous system lesions. Notably, peripheral blood is a practical and repeatable sampling site that captures circulating immune cells and their epigenetic state, which is particularly relevant for MS, where peripheral B-cell abnormalities are well established; for BD, the same tissue compartment is less directly coupled to the neuropsychiatric phenotype, likely explaining the lower AUC. As a peripheral blood biomarker with minimal sampling trauma, *BRD4* may alleviate two prominent clinical challenges: frequent misdiagnosis of bipolar disorder and the lack of reliable early molecular indicators for multiple sclerosis. Nevertheless, multi-variable predictive nomograms integrating clinical metadata and immune cell abundance metrics are required to further improve overall discriminatory accuracy in future translational research.

This study has several notable limitations that constrain the generalizability of our mechanistic inferences, which we acknowledge objectively as follows. First, all transcriptional data were retrieved from public microarray repositories with relatively small sample sizes, and unified clinical covariate information was unavailable to eliminate transcriptional confounding effects; the BD external validation cohort in particular comprised only eight cases, and its wide confidence interval should be interpreted cautiously. Second, we only verified transcriptional upregulation of *BRD4* in patient cohorts at the mRNA level; its upstream epigenetic regulators, downstream transcriptional regulatory networks, and direct biological functions remain to be confirmed via cellular and animal experimental models, and we cannot equate mRNA expression levels to corresponding protein abundance or epigenetic activity. Third, all biospecimens analyzed were peripheral blood and peripheral blood mononuclear cells, leaving *BRD4* expression patterns and regulatory activity within central nervous system tissue uncharacterized; single-cell transcriptomics would be needed to disentangle which cell types drive the observed *BRD4* signal. Fourth, the predictive nomogram constructed in this work only incorporates *BRD4* as a single predictive variable without integrating clinical or neuroimaging indicators, which restricts its overall discriminatory capacity. Fifth, CIBERSORT deconvolution estimates are inferred rather than directly measured, and the LM22 signature matrix was originally trained on tumor samples; cell-type proportions should therefore be treated as approximate and ideally validated by flow cytometry.

Future research can be advanced from three complementary dimensions. For bioinformatic extension, multi-omics datasets including DNA methylation and single-cell transcriptomics will be integrated to map *BRD4*-centered ceRNA and epigenetic regulatory networks, with validation conducted in larger independent clinical cohorts. For basic laboratory verification, *BRD4* overexpression and knockdown cell lines, together with bromodomain and extra-terminal (BET) family inhibitor intervention experiments, will be established to verify its modulatory effects on inflammation and synaptic function; BD and MS animal models will further clarify the in vivo role of *BRD4* in neuroinflammation and demyelinating pathology. For clinical translational research, prospective cohorts covering healthy controls, isolated BD patients, isolated MS patients, and patients with comorbid BD-MS will be recruited to determine the optimal *BRD4* expression cut-off value for auxiliary clinical screening and optimize multi-factor predictive nomograms. Additionally, BET inhibitors, which block *BRD4*’s acetylated-histone-binding pocket and thereby suppress its transcriptional co-activator function, deserve further investigation as dual-targeted therapeutic agents to alleviate mood dysfunction in BD and demyelinating injury in MS, as well as overlapping clinical manifestations in comorbid patients. Brain-permeable selective BET inhibitors have been validated to simultaneously suppress microglial overactivation and restore synaptic function in preclinical neuropsychiatric and demyelination models [32].

The present work contains three core innovations. First, we constructed an integrated multi-layer pipeline combining DEG screening, WGCNA, PPI network clustering, and three independent machine learning algorithms, and identified *BRD4* as the unique shared epigenetic hub gene of BD and MS. Second, we systematically delineated the *BRD4*-mediated immune-neural crosstalk axis that may underlie convergent pathological mechanisms of BD and MS. Third, we validated peripheral blood *BRD4* as a non-invasive auxiliary screening biomarker, and it represents a candidate therapeutic target warranting further investigation for both diseases. Collectively, this study establishes a multi-dimensional bioinformatic screening framework and reports *BRD4* as a shared core epigenetic gene associated with BD and MS pathogenesis. *BRD4* is significantly upregulated in peripheral blood samples from both patient populations with consistent predictive capacity across independent cohorts, and it is associated with immune-neural signaling crosstalk in BD and MS. These findings expand our understanding of convergent molecular mechanisms linking severe psychiatric disease and central autoimmune disorders, and support the hypothesis that *BRD4* may serve as a cross-disease auxiliary diagnostic biomarker and candidate therapeutic target for bipolar disorder and multiple sclerosis.

## 5. Conclusions

This study, employing a multi-dimensional screening strategy integrating DEG analysis, WGCNA, PPI network analysis, and machine learning, identifies *BRD4* as a shared core gene between BD and MS. *BRD4* is highly expressed in both disorders and exhibits reliable diagnostic efficacy, and may participate in the pathological processes of BD and MS by regulating “immune-neural” crosstalk pathways. These findings provide a novel molecular perspective for understanding the comorbidity mechanisms between psychiatric disorders and autoimmune diseases, and *BRD4* shows promise as a cross-disease auxiliary diagnostic biomarker for BD and MS.

## Figures and Tables

**Table 1:** Basic information of the included gene expression datasets.

Dataset ID	Disease Type	Sample Type	Platform	Sample Size (Case/Ctrl)
GSE23848	BD	Peripheral Blood RNA	GPL6106	20/15
GSE21942	MS	PBMC RNA	GPL570	12/15
GSE39653	BD (Validation)	Peripheral Blood RNA	GPL10558	8/24
GSE203241	MS (Validation)	PBMC RNA	GPL571	38/21

***Note:** case/control counts reflect the final analytic samples after excluding unrelated diagnostic groups. Age and sex variables were not deposited with the GEO metadata for these cohorts.*

## Data Availability

The data used in this study are publicly available in the GEO database (https://www.ncbi.nlm.nih.gov/geo/) under accession numbers: GSE23848, GSE21942, GSE39653, and GSE203241. The code supporting the findings of this study are available from the corresponding author upon reasonable request.

## Supplementary Materials

The following supporting information can be downloaded at: https://www.mdpi.com/article/10.3390/cimb48101077/s1.

## Abbreviations

The following abbreviations are used in this manuscript:

BD	Bipolar Disorder
MS	Multiple Sclerosis
DEG	Differentially Expressed Gene
WGCNA	Weighted Gene Co-expression Network Analysis
SCM	Signature Co-expression Module gene
PPI	Protein–Protein Interaction
LASSO	Least Absolute Shrinkage and Selection Operator
RF	Random Forest
SVM-RFE	Support Vector Machine-Recursive Feature Elimination
ROC	Receiver Operating Characteristic
AUC	Area Under Curve
DCA	Decision Curve Analysis
GEO	Gene Expression Omnibus
PBMC	Peripheral Blood Mononuclear Cell
GO	Gene Ontology
KEGG	Kyoto Encyclopedia of Genes and Genomes
MCODE	Molecular Complex Detection
CI	Confidence Interval
CRediT	Contributor Roles Taxonomy
