# Supplementary material for: Identification of BRD4 as a Shared Diagnostic Biomarker Between Bipolar Disorder and Multiple Sclerosis Using Machine Learning
Source: Curr Issues Mol Biol. 2026 Oct 19;48(10):1077. doi: 10.3390/cimb48101077 (PMC13702881; doi:10.3390/cimb48101077)
Supplement: Supplementary file 1 [file cimb-48-01077-s001.zip › cimb-4565732-supplementary.pdf]

Table S1a. Candidate genes screened by LASSO, random forest and SVM-RFE in the MS discovery cohort (GSE21942).

Algorithm	Number of genes	Candidate gene list
LASSO	8	FGD2, RPS24, SFPQ, BRD4, TNFAIP3, NDUFB2, YWHAG, SIN3A
Random Forest	10	BRD4, NDUFB2, SFPQ, SIN3A, YWHAG, NDUFS3, RBBP4, NT5E, COX7B, EEF1D
SVM-RFE	27	YWHAG, FGD2, SIN3A, COX6C, TNFAIP3, RPS24, NDUFB2, NT5E, SFPQ, BRD4, RBM5, RBBP4, CD47, EEF1D, HDAC2, PPP1CA, SRP72, BRD1, CERK, HAVCR2, TRAM1, COX7B, CYBB, AURKAIP1, PFDN5, NDUFC2, SSR2

Table S1b. Candidate genes screened by LASSO, random forest and SVM-RFE in the BD discovery cohort (GSE23848).

Algorithm	Number of genes	Candidate gene list
LASSO	11	SRP72, CERK, BRD4, EIF4A2, RPL26L1, SRP14, RHOB, AURKAIP1, SGPP1, DDX5, RBM25
Random Forest	9	NDUFA1, NEDD8, AURKAIP1, RPL26L1, YWHAG, COX7B, BRD4, RHOB, PPP1R12A
SVM-RFE	26	RPL26L1, RBM25, SRP14, RHOB, EIF4A2, COX7B, DDX5, NDUFA1, MAP3K1, SGPP1, BRD4, METAP2, AURKAIP1, CERK, NEDD8, UQCRC1, PSIP1, SRP72, FGD2, NDUFS3, COX6C, RPS27L, RPS24, TNFAIP3, EEF1D, RBBP4

Table S2. Discriminative performance of BRD4 across the four cohorts. AUC with 95% CI was calculated by pROC with 1000 bootstrap resamples; sensitivity and specificity were derived at the optimal Youden threshold.

Cohort	AUC (95% CI)	Sensitivity	Specificity
GSE23848(BD discovery)	0.713 (0.54 – 0.89)	0.95	0.60
GSE21942(MS discovery)	0.998 (0.99 – 1.00)	1.00	0.93
GSE39653(BD validation)	0.775 (0.59 – 0.96)	0.50	0.92
GSE203241(MS validation)	0.828 (0.71 – 0.93)	0.92	0.62

Table S3a. CIBERSORT-estimated immune cell fractions in the BD cohort (GSE23848): median fraction in BD patients and controls, Wilcoxon rank-sum P values.

Immune cell type	BD median	Control median	Wilcoxon P	Sig.
T cells follicular helper	0.129	0.163	0.0277	*
B cells naive	0.061	0.0304	0.0613	ns
B cells memory	0.104	0.0695	0.0994	ns
Dendritic cells resting	0.0000627	0	0.1016	ns
Eosinophils	0	0.0037	0.1071	ns
NK cells activated	0.0496	0.0558	0.1133	ns
T cells regulatory (Tregs)	0.0335	0.00816	0.1436	ns
T cells gamma delta	0.0454	0	0.1618	ns
Plasma cells	0	0.00494	0.1695	ns
Macrophages M0	0	0	0.2297	ns
T cells CD8	0.208	0.243	0.2827	ns
Neutrophils	0.0949	0.0908	0.3467	ns
T cells CD4 memory resting	0	0	0.4189	ns
Dendritic cells activated	0	0	0.4743	ns
NK cells resting	0	0	0.5407	ns
Macrophages M2	0	0.00593	0.5589	ns
Macrophages M1	0.039	0.0344	0.5644	ns
T cells CD4 memory activated	0.099	0.00815	0.7873	ns
Monocytes	0.181	0.153	0.8051	ns
Mast cells activated	0	0	0.9011	ns
Mast cells resting	0.0517	0.0465	0.9345	ns
T cells CD4 naive	0	0	NaN	NA

Table S3b. Pearson correlation between BRD4 expression and each immune cell type in the BD cohort (GSE23848).

Immune cell type	Pearson r	P value	Sig.
T cells CD4 memory resting	0.379	0.0246	*
Macrophages M2	-0.281	0.1018	ns
NK cells activated	-0.239	0.1665	ns
Macrophages M1	0.236	0.1717	ns
T cells follicular helper	-0.233	0.1777	ns
Plasma cells	-0.198	0.2538	ns
Monocytes	0.144	0.4105	ns
T cells CD8	0.144	0.4106	ns
Dendritic cells resting	0.132	0.449	ns
Dendritic cells activated	0.0948	0.5881	ns
B cells naive	-0.0894	0.6096	ns
Eosinophils	0.0884	0.6137	ns
B cells memory	0.068	0.698	ns
Mast cells resting	0.0679	0.6985	ns
T cells CD4 memory activated	-0.0344	0.8444	ns
T cells gamma delta	-0.0319	0.8557	ns

Macrophages M0	0.0234	0.8937	ns
T cells regulatory (Tregs)	0.0227	0.8968	ns
Mast cells activated	-0.0183	0.9167	ns
Neutrophils	-0.0181	0.9179	ns
NK cells resting	-0.00297	0.9865	ns
T cells CD4 naive	NA	NA	NA

Table S3c. CIBERSORT-estimated immune cell fractions in the MS cohort (GSE21942): median fraction in MS patients and controls, Wilcoxon rank-sum P values.

Immune cell type	MS median	Control median	Wilcoxon P	Sig.
B cells naive	0.051	0.00931	0.0000192	***
Eosinophils	0.058	0.04274	0.000765	***
Plasma cells	0.0103	0.02213	0.0157	*
Mast cells resting	0.0345	0.02026	0.0157	*
T cells CD4 naive	0.0731	0.03812	0.0202	*
T cells CD8	0.0964	0.11819	0.0511	ns
Neutrophils	0.0455	0.04161	0.112	ns
Monocytes	0.2212	0.26523	0.134	ns
Dendritic cells resting	0	0.006209	0.139	ns
T cells CD4 memory resting	0.0551	0.08287	0.217	ns
Macrophages M1	0	0.000726	0.231	ns
T cells regulatory (Tregs)	0	0	0.305	ns
B cells memory	0.0546	0.0413	0.326	ns
NK cells resting	0.1537	0.16327	0.4	ns
Macrophages M0	0.018	0.0195	0.484	ns
T cells CD4 memory activated	0.056	0.05709	0.591	ns
Macrophages M2	0.0192	0.02021	0.591	ns
Dendritic cells activated	0	0.000513	0.627	ns
NK cells activated	0	0	0.68	ns
T cells gamma delta	0.0292	0.03513	0.809	ns
T cells follicular helper	NA	NA	NA	NA
Mast cells activated	NA	NA	NA	NA

Table S3d. Pearson correlation between BRD4 expression and each immune cell type in the MS cohort (GSE21942).

Immune cell type	Pearson r	P value	Sig.
B cells naive	0.844	0.000000009	***
Eosinophils	0.522	0.00364	**
Monocytes	-0.381	0.0417	*
T cells CD4 naive	0.358	0.0564	ns
T cells CD8	-0.35	0.0629	ns
Neutrophils	0.311	0.101	ns
Dendritic cells resting	-0.301	0.113	ns

Plasma cells	-0.251	0.19	ns
Macrophages M0	0.229	0.231	ns
Mast cells resting	0.203	0.292	ns
T cells CD4 memory resting	-0.184	0.341	ns
T cells regulatory (Tregs)	-0.133	0.493	ns
NK cells resting	-0.13	0.5	ns
Macrophages M1	-0.11	0.571	ns
T cells CD4 memory activated	0.109	0.572	ns
B cells memory	-0.108	0.577	ns
Dendritic cells activated	0.103	0.595	ns
NK cells activated	0.0565	0.717	ns
Macrophages M2	0.0015	0.994	ns
T cells gamma delta	0.0004	0.998	ns
T cells follicular helper	NA	NA	NA
Mast cells activated	NA	NA	NA

Figure S1a. Soft-threshold selection for BD WGCNA.

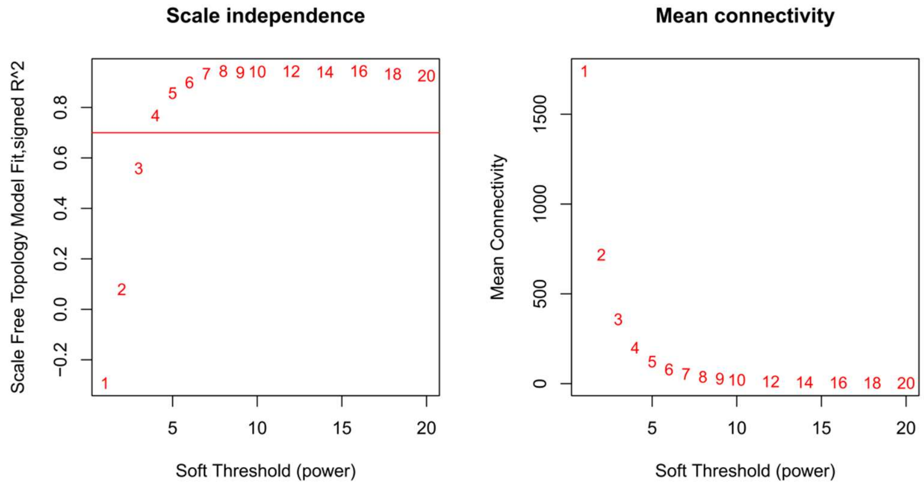

Figure S1b. Gene dendrogram and modules, BD cohort.

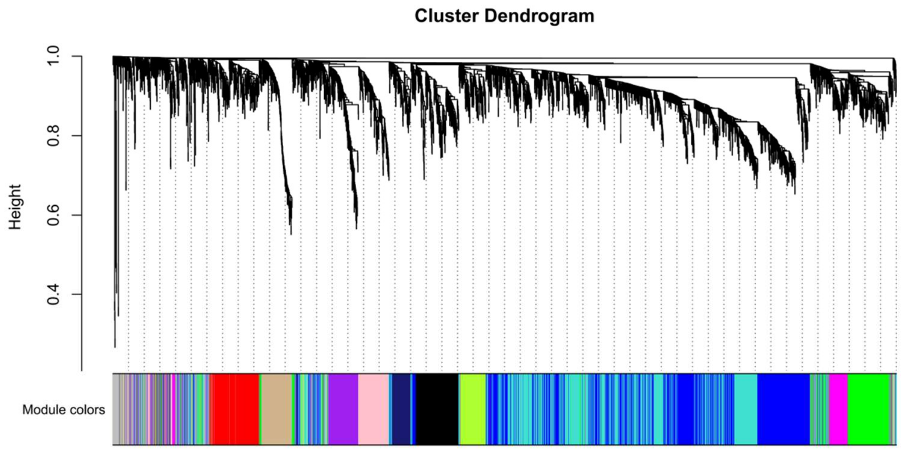

Figure S1c. Soft-threshold selection for MS WGCNA.

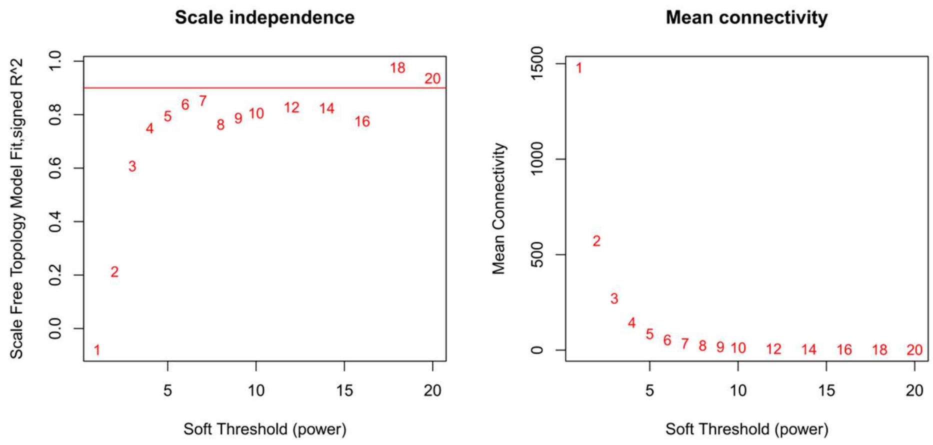

Figure S1d. Gene dendrogram and modules, MS cohort.

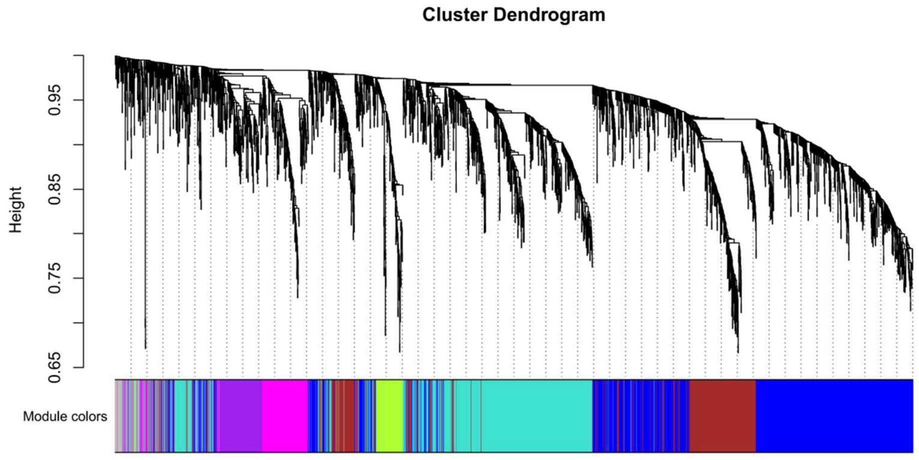

Figure S1. WGCNA diagnostics. (a) Soft-threshold power selection for the BD network; (b) gene dendrogram and module colors for BD; (c) soft-threshold power selection for the MS network; (d) gene dendrogram and module colors for MS.
